# Supplementary material for: Association between red blood cell distribution width-to-albumin ratio at admission and all-cause mortality in patients with acute pancreatitis based on the MIMIC-III database
Source: PLoS One. 2025 Feb 7;20(2):e0318873. doi: 10.1371/journal.pone.0318873 (PMC11805432; doi:10.1371/journal.pone.0318873)
Supplement: S1 Table — (PDF) [file pone.0318873.s001.pdf]

Supplementary materials

Supplementary Table 1

Collinearity analysis showed that all variance inflation factors of included variables were lower than 10, indicating that no collinearity existed among the variables.

Supplementary materials Table 1. Collinearity analysis

	P-value	Tolerance	Variance Inflation Factor
Ethnicity	0.956	0.961	1.041
Congestive heart failure	0.173	0.865	1.156
Hypertension	0.114	0.789	1.268
Chronic pulmonary disease	0.059	0.916	1.091
Diabetes	0.161	0.919	1.088
Renal failure	0.895	0.819	1.221
Mechanical ventilation	0.056	0.648	1.544
Age	0.606	0.539	1.854
Weight	0.760	0.842	1.188
SAPSII	0.574	0.280	3.577
SOFA	0.738	0.339	2.953
Heart rate	0.876	0.619	1.615
Bp	0.323	0.754	1.326
Respiratory rate	0.534	0.747	1.338
Temperature	0.693	0.720	1.389
Urine output	0.566	0.807	1.239
Potassium	0.039	0.841	1.190
RAR	0.195	0.234	4.274
Tb	0.000	0.849	1.178
Creatinine	0.277	0.475	2.105
Bun	0.003	0.477	2.094
Lactate	0.000	0.833	1.200
Hb	0.321	0.403	2.481
Glucose	0.237	0.859	1.164
Platelet	0.231	0.801	1.248
Alt	0.677	0.811	1.233
Hematocrit	0.230	0.438	2.282
PT	0.481	0.177	5.639
PTT	0.006	0.796	1.256
Neut	0.075	0.862	1.160

INR	0.932	0.179	5.594
Gender	0.941	0.869	1.151

Abbreviations: SAPSII, simplified acute physiology score II; SOFA, sequential organ failure assessment; Bp, blood pressure; RAR, red blood cell distribution width-to-albumin ratio; Tb, total bilirubin; Bun, blood urea nitrogen; Hb, Hemoglobin; Alt, alanine aminotransferase; PT, prothrombin time; PTT, partial thromboplastin time; Neut, neutrophil; INR, International Normalized Ratio.
