# Supplementary material for: Association between red blood cell distribution width-to-albumin ratio at admission and all-cause mortality in patients with acute pancreatitis based on the MIMIC-III database
Source: PLoS One. 2025 Feb 7;20(2):e0318873. doi: 10.1371/journal.pone.0318873 (PMC11805432; doi:10.1371/journal.pone.0318873)
Supplement: S2 Table — (PDF) [file pone.0318873.s002.pdf]

Supplementary Table 2

Baseline characteristics of patients grouped by the survival status at 28 days

Characteristic	status		p-value
	Survival(n = 822)	Non-survival(n = 109)	
Age (years)	58 (46, 71)	61 (50, 72)	0.134
Weight (kg)	83 (73, 86)	83 (73, 86)	0.575
SAPSII	36 (26, 46)	35 (27, 44)	0.799
SOFA	5.0 (3.0, 8.0)	4.0 (2.0, 6.0)	0.069
Heart rate (times/min）	94 (81, 108)	92 (80, 104)	0.359
Bp (mm Hg)	80 (73, 90)	79 (72, 88)	0.254
Respiratory rate (times/min)	20.5 (17.3, 23.5)	19.5 (16.5, 22.0)	0.048
Temperature (℃)	37.05 (36.60, 37.51)	36.90 (36.56, 37.32)	0.203
Urine output (ml)	1,641 (995, 2,548)	1,655 (1,015, 2,395)	0.614
Potassium(mg/dL)	4.15 (3.80, 4.15)	4.15 (3.80, 4.70)	0.015
Tb (mg/dL)	0.80 (0.50, 2.10)	1.70 (0.60, 5.10)	<0.001
Creatinine (mg/dL)	1.10 (0.80, 1.80)	1.60 (1.00, 2.60)	<0.001
Bun (mg/dL)	21 (13, 34)	35 (21, 51)	<0.001
Lactate (mmol/L)	2.10 (1.30, 2.66)	2.10 (1.50, 4.50)	0.017
Hb (mg/dL)	12.00 (10.40, 13.70)	11.20 (10.10, 13.10)	0.014
Glucose (mg/dL)	130 (103, 173)	140 (107, 187)	0.093
Platelet (10^9/L)	226 (164, 311)	195 (113, 290)	0.002
Alt (U/L)	42 (21, 129)	60 (25, 176)	0.035
Hematocrit (mm/h)	30.6 (26.4, 34.7)	29.5 (25.3, 33.1)	0.032
PT (s)	15.0 (13.5, 17.3)	16.3 (13.9, 19.9)	<0.001
PTT (s)	33 (28, 40)	39 (29, 59)	<0.001
Neut(10^9/L)	12 (8, 15)	12 (9, 18)	0.017
INR	1.40 (1.20, 1.75)	1.60 (1.20, 2.10)	<0.001
Ethnicity, n(%)			0.444
White	554 (67.4%)	68 (62.4%)	
Black	77 (9.4%)	14 (12.8%)	
Asian	25 (3.0%)	2 (1.8%)	
Hispanic OR Latino	26 (3.2%)	6 (5.5%)	
Other	140 (17.0%)	19 (17.4%)	
Congestive heart failure, n(%)			0.208
No	633 (77.0%)	78 (71.6%)	
Yes	189 (23.0%)	31 (28.4%)	
Hypertension, n (%)			0.150

No	392 (47.7%)	44 (40.4%)	
Yes	430 (52.3%)	65 (59.6%)	
Chronic pulmonary disease, n(%)			0.116
No	683 (83.1%)	97 (89.0%)	
Yes	139 (16.9%)	12 (11.0%)	
Diabetes, n (%)			0.026
No	644 (78.3%)	75 (68.8%)	
Yes	178 (21.7%)	34 (31.2%)	
Renal failure, n (%)			0.435
No	709 (86.3%)	91 (83.5%)	
Yes	113 (13.7%)	18 (16.5%)	
Mechanical ventilation, n (%)			0.037
No	441 (53.6%)	70 (64.2%)	
Yes	381 (46.4%)	39 (35.8%)	
Gender, n (%)			0.076
Male	466 (56.7%)	52 (47.7%)	
Female	356 (43.3%)	57 (52.3%)	
RAR group			<0.001
Low RAR	354 (43.1%)	19 (17.4%)	
High RAR	468 (56.9%)	90 (82.6%)	

Abbreviations: SAPSII, simplified acute physiology score II; SOFA, sequential organ failure assessment; Bp, blood pressure; Tb, total bilirubin; Bun, blood urea nitrogen; Hb, Hemoglobin; Alt, alanine aminotransferase; PT, prothrombin time; PTT, partial thromboplastin time; Neut, neutrophil; INR, International Normalized Ratio.
