# Supplementary material for: Association between red blood cell distribution width-to-albumin ratio at admission and all-cause mortality in patients with acute pancreatitis based on the MIMIC-III database
Source: PLoS One. 2025 Feb 7;20(2):e0318873. doi: 10.1371/journal.pone.0318873 (PMC11805432; doi:10.1371/journal.pone.0318873)
Supplement: S3 Table — (PDF) [file pone.0318873.s003.pdf]

Supplementary Table 3

Univariate and multivariable cox regression analysis of factors influencing 28-day mortality.

Variables	Univariate cox regression					Multivariable cox regression				
	β	S.E	Z	P	HR (95%CI)	β	S.E	Z	P	HR (95%CI)
Age	0.01	0.01	1.52	0.127	1.01 (1.00 ~ 1.02)					
Weight	-0.00	0.01	-0.85	0.396	1.00 (0.99 ~ 1.01)					
SAPSII	-0.00	0.01	-0.71	0.477	1.00 (0.98 ~ 1.01)					
SOFA	-0.06	0.03	-2.00	0.045	0.95 (0.90 ~ 0.99)	0.01	0.03	0.19	0.851	1.01 (0.95 ~ 1.07)
Heart rate	-0.00	0.01	-0.74	0.459	1.00 (0.99 ~ 1.01)					
Bp	-0.01	0.01	-1.07	0.285	0.99 (0.98 ~ 1.01)					
Respiratory rate	-0.04	0.02	-1.70	0.089	0.96 (0.92 ~ 1.01)					
Temperature	-0.13	0.13	-0.98	0.328	0.88 (0.68 ~ 1.14)					
Urine output	-0.00	0.00	-1.02	0.308	1.00 (1.00 ~ 1.00)					
Potassium	0.27	0.08	3.20	0.001	1.31(1.11 ~ 1.54)	0.25	0.11	2.35	0.019	1.28(1.04 ~ 1.58)
Tb	0.08	0.01	8.04	<.001	1.08(1.06~1.10)	0.07	0.01	5.06	<.001	1.07(1.04~1.10)
Creatinine	0.10	0.04	2.73	0.006	1.10(1.03~1.18)	-0.04	0.05	-0.80	0.426	0.96(0.87~1.06)
Bun	0.01	0.00	4.64	<.001	1.01(1.01~1.01)	0.01	0.00	3.29	<.001	1.01(1.01~1.02)
Lactate	0.13	0.02	5.47	<.001	1.14(1.09~1.20)	0.11	0.03	3.62	<.001	1.11(1.05~1.18)
Hb	-0.08	0.04	-2.02	0.044	0.92(0.86~0.99)	-0.05	0.06	-0.76	0.446	0.95(0.84~1.08)
Glucose	0.00	0.00	1.16	0.247	1.00(1.00~1.00)					
Platelet	-0.01	0.00	-2.60	0.009	0.99(0.99~0.99)	-0.00	0.00	-1.43	0.152	1.00(1.00~1.00)
Alt	0.00	0.00	0.43	0.668	1.00(1.00~1.00)					
Hematocrit	-0.03	0.02	-2.19	0.028	0.97(0.94~0.99)	0.02	0.03	0.80	0.426	1.02(0.97~1.07)
PT	0.01	0.01	1.41	0.159	1.01(1.00~1.03)					
PTT	0.01	0.00	5.55	<.001	1.01(1.01~1.02)	0.01	0.00	2.51	0.012	1.01(1.01~1.01)
Neut	0.02	0.01	2.42	0.016	1.02(1.01~1.04)	0.02	0.01	2.09	0.036	1.02(1.01~1.04)
INR	0.09	0.05	1.91	0.056	1.10(1.00~1.20)					
RAR group										
Low RAR					1.00(Reference)					
High RAR	1.22	0.25	4.82	<.001	3.37(2.06~5.54)	0.96	0.27	3.55	<.001	2.62(1.54~4.45)
Ethnicity										
White					1.00(Reference)					
Black	0.38	0.29	1.28	0.199	1.46(0.82~2.59)					
Asian	-0.42	0.72	-0.59	0.556	0.66(0.16~2.68)					
Hispanic OR Latino	0.54	0.43	1.26	0.206	1.71(0.74~3.95)					
Other	0.10	0.26	0.39	0.697	1.11(0.67~1.84)					
Congestive heart failure										
No					1.00(Reference)					
Yes	0.27	0.21	1.27	0.203	1.31(0.86~1.99)					
Hypertension										

No					1.00(Reference)					
Yes	0.29	0.20	1.48	0.138	1.34(0.91~1.96)					
Chronic pulmonary disease										
No					1.00(Reference)					
Yes	-0.48	0.31	-1.58	0.114	0.62(0.34~1.12)					
Diabetes										
No					1.00(Reference)					1.00(Reference)
Yes	0.47	0.21	2.26	0.024	1.60(1.06~2.39)	0.33	0.21	1.57	0.117	1.39(0.92~2.11)
Renal failure										
No					1.00(Reference)					
Yes	0.20	0.26	0.78	0.437	1.22(0.74~2.03)					
Mechanical Ventilation										
No					1.00(Reference)					
Yes	-0.41	0.20	-2.03	0.042	0.67(0.45~0.99)	-0.51	0.22	-2.27	0.023	0.60(0.39~0.93)
Gender										
Male					1.00(Reference)					
Female	0.33	0.19	1.70	0.090	1.38(0.95~2.02)					

Abbreviations: SAPSII, simplified acute physiology score II; SOFA, sequential organ failure assessment; Bp, blood pressure; Tb, total bilirubin; Bun, blood urea nitrogen; Hb, Hemoglobin; Alt, alanine aminotransferase; PT, prothrombin time; PTT, partial thromboplastin time; Neut, neutrophil; INR, International Normalized Ratio; HR, Hazards Ratio; CI, Confidence Interval.
