# Supplementary material for: Association between red blood cell distribution width-to-albumin ratio at admission and all-cause mortality in patients with acute pancreatitis based on the MIMIC-III database
Source: PLoS One. 2025 Feb 7;20(2):e0318873. doi: 10.1371/journal.pone.0318873 (PMC11805432; doi:10.1371/journal.pone.0318873)
Supplement: Attachment — Submitted filename: review.docx [file pone.0318873.s004.docx]

1. Statistical and Methodological Issues

Handling of Missing Data:

The article mentions the use of multiple imputation to handle missing data but does not clearly describe the imputation methods and steps. It should be clarified whether appropriate imputation models (e.g., regression-based or machine learning methods) were used.

It is recommended to explicitly report the proportion and distribution of missing data, especially for key variables like RAR-related metrics, to determine if there is systematic missingness.

Rationale for Grouping:

RAR was divided into quartiles for grouping, which, while convenient, may obscure potential nonlinear associations. Was the sensitivity of the results to grouping strategies (e.g., logarithmic partitioning or other clinically relevant thresholds) evaluated?

Multicollinearity of Variables:

Although the article mentions evaluating multicollinearity, it does not clearly explain which variables were adjusted or excluded. It is recommended to further elaborate in the results section on how multicollinearity issues were addressed, especially for interrelated variables such as Hb, BUN, and PT.

2. Interpretation of Results and Logical Consistency

Kaplan-Meier Analysis:

The Kaplan-Meier curves show significant differences in 28-day and 60-day survival rates, but the lack of significant difference between Q2 and Q3 at 28 days (p = 0.366) is not fully explained. This could indicate minor biological differences between these groups and should be further discussed.

Cox Regression Model:

The Cox regression analysis identifies RAR as a significant risk factor but does not sufficiently explain the logic for selecting adjustment variables. If potential confounders were not included in the model, this might affect the conclusions.

The results section does not mention whether the proportional hazards assumption was tested. It should be clarified if this assumption was met.

3. Clinical Interpretation and Applicability

Clinical Significance:

Although RAR is statistically significant, its actual clinical relevance has not been adequately explored. For instance, does RAR significantly improve the predictive power of existing scoring systems like SOFA or APACHE-II? A comparative analysis using ROC curves is suggested.

Comparison with Existing Studies:

The article mentions differences between RAR and other indicators (e.g., CRP/lymphocyte ratio) but does not discuss RAR's specific advantages (e.g., cost, ease of measurement). Are there direct comparative data available?

4. Potential Language and Expression Issues

Ambiguous Statements:

For example: "A significant positive correlation was noticed between RAR quartiles and all-cause mortality (p < 0.001)".

This statement is overly broad. It is recommended to specify what "significant" means in terms of effect size or HR range.

Suggested revision: "RAR quartiles demonstrated a dose-response relationship with all-cause mortality, with a hazard ratio of up to X.X in the highest quartile (p < 0.001)."

Inconsistent Terminology:

Terms such as "RAR" are sometimes referred to as a ratio and at other times as an index. Consistency should be maintained throughout the article, and its definition should be clarified when first introduced.

5. Potential Data Issues

Baseline Characteristics Imbalance:

Some baseline characteristics (e.g., ALT, PTT) show significant differences across groups in Table 1, but the article does not discuss their potential influence. It is recommended to address the potential biases introduced by these differences.

Lack of External Validation:

Reliance on a single database (MIMIC-III) limits the generalizability of the results. Mentioning the need for future validation with independent datasets in the discussion would strengthen the study.

6. Technical Details

ROC Analysis:

The reported AUC of 0.669, though statistically significant, indicates limited predictive ability. This should be acknowledged in the discussion, and the potential for combining RAR with other indicators to enhance predictive power should be explored.

Adjustment of Variables:

The article mentions adjusting for all variables with p < 0.05, but the comprehensiveness and methodology of the adjustments are unclear. For example, was the role of gender and other potential confounders thoroughly addressed?

7. Formatting and Citation Issues

Text Formatting:

Ensure that the reference style fully complies with journal requirements.

Citation Count:

The number of references appears to be relatively low. Consider adding citations to fully support the content of the article.

Conclusion:

The overall conclusions of the article are reliable, but there is room for improvement in the transparency of methods, interpretation of results, and discussion of clinical relevance. Addressing the above issues can significantly enhance the scientific quality and impact of the article.
