# Supplementary material for: Association between red blood cell distribution width-to-albumin ratio at admission and all-cause mortality in patients with acute pancreatitis based on the MIMIC-III database
Source: PLoS One. 2025 Feb 7;20(2):e0318873. doi: 10.1371/journal.pone.0318873 (PMC11805432; doi:10.1371/journal.pone.0318873)
Supplement: Attachment — Submitted filename: Response to Reviewers.doc [file pone.0318873.s005.doc]

Dear Editors and Reviewers:

Thanks for your letter and for the comments concerning our manuscript entitled "Association between red blood cell distribution width-to-albumin ratio at admission and all-cause mortality in patients with acute pancreatitis based on the MIMIC-III database"(ID:PONE-D-24-38811).Those comments are all very valuable and helpful for revising and improving our paper,as well as the important guiding significance to our researches. We have made corresponding correction based on the comments. Revised portion are marked in red in the paper. The main corrections in the paper and the responds to the reviewer's comments are as follows:

**1.Response to comment:** The article mentions the use of multiple imputation to handle missing data but does not clearly describe the imputation methods and steps. It should be clarified whether appropriate imputation models (e.g., regression-based or machine learning methods) were used.

**Response:** We are very sorry for our negligence of unclearly describing the imputation methods and steps. After revision, specific methods and steps have been added to the article.We performed these operations by using the mice package in R software and carried out the imputation through regression-based models.

**2.Response to comment:** It is recommended to explicitly report the proportion and distribution of missing data, especially for key variables like RAR-related metrics, to determine if there is systematic missingness.

**Response:** After the data extraction was completed, a missing value analysis was conducted, and variables with more than 20% missing values were deleted, including pH, PCO2, PO2, and CRP. Additionally, cases with missing RAR, which is either RDW or ALB (a total of 3 cases), were excluded.

**3.Response to comment:** RAR was divided into quartiles for grouping, which, while convenient, may obscure potential nonlinear associations. Was the sensitivity of the results to grouping strategies (e.g., logarithmic partitioning or other clinically relevant thresholds) evaluated?

**Response:** The concern raised by the reviewers about the potential nonlinear associations by the quartile grouping of the RAR variable is one that we take very seriously. Here is our analysis and adjustment of this issue:The choice to use quartile grouping was based on its ability to simplify the interpretation of the data and to make it more accessible for clinicians to understand and apply. However, we fully acknowledge that this grouping strategy may fail to capture nonlinear relationships between variables. To address this, we have adjusted our grouping strategy. First, we conducted a ROC analysis to determine the cutoff value, and then we grouped the patients based on this cutoff value. The cutoff value for this study is 4.39, which is similar to that identified in previous study (4.35). Additionally, we utilized restricted cubic splines to explore the nonlinear relationship between RAR and survival time. The results indicated that there was no nonlinear relationship between RAR and survival time.

**4.Response to comment:** Although the article mentions evaluating multicollinearity, it does not clearly explain which variables were adjusted or excluded. It is recommended to further elaborate in the results section on how multicollinearity issues were addressed, especially for interrelated variables such as Hb, BUN, and PT.

**Response:** In the results section of the article, it is mentioned that the variance inflation factor method was used to assess multicollinearity among variables. As seen in the supplementary materials, the collinearity analysis results indicated that all variance inflation factors for the included variables were less than 10, suggesting that there was no multicollinearity among the variables. Therefore, no variable packages were excluded in the final analysis.

**5.Response to comment:** The Kaplan-Meier curves show significant differences in 28-day and 60-day survival rates, but the lack of significant difference between Q2 and Q3 at 28 days (p = 0.366) is not fully explained. This could indicate minor biological differences between these groups and should be further discussed.

**Response:** Due to the addition of Propensity Score Matching and Restricted Cubic Spline analyses, the content of the article has increased significantly. Therefore, after revision, the article focuses solely on the impact of RAR on the 28-day mortality of acute pancreatitis. The groups have been reduced from four to two. After revision, the Kaplan-Meier Analysis showed that the 28-day mortality of patients in the high RAR group was significantly increased compared to the low RAR group in both the unmatched cohort and the propensity score-matched cohort.

**6.Response to comment:** The Cox regression analysis identifies RAR as a significant risk factor but does not sufficiently explain the logic for selecting adjustment variables. If potential confounders were not included in the model, this might affect the conclusions.

**Response:** Regarding the selection of adjustment variables, in model 2, the adjustment variables are demographic characteristics. Model 3 builds upon Model 2 by adding variables with a p-value less than 0.05 in the multivariate analysis, including potassium, total bilirubin, blood urea nitrogen, lactate, partial thromboplastin time, and neutrophil count. Model 4 further includes the use of invasive mechanical ventilation on basis of the variables included in Model 3, in an effort to encompass as many potential confounders as possible.

**7.Response to comment:** The results section does not mention whether the proportional hazards assumption was tested. It should be clarified if this assumption was met.

**Response:** Before conducting the Cox regression analysis, the proportional hazards assumption was assessed using the Schoenfeld residuals method. The results indicated that all independent variables satisfied the proportional hazards assumption. The aforementioned content has been added to study outcomes section in the article.

**8.Response to comment:** Although RAR is statistically significant, its actual clinical relevance has not been adequately explored. For instance, does RAR significantly improve the predictive power of existing scoring systems like SOFA or APACHE-II? A comparative analysis using ROC curves is suggested.

**Response:** The data for this study were sourced from the MIMIC-III database, which didn’t include APACHE-II scores. This study found that the AUC of RAR was higher than that of SOFA, indicating that RAR had predictive value for the prognosis of patients with acute pancreatitis.

**9.Response to comment:** The article mentions differences between RAR and other indicators (e.g., CRP/lymphocyte ratio) but does not discuss RAR's specific advantages (e.g., cost, ease of measurement). Are there direct comparative data available?
**Response:** After reviewing the literature, there are no detailed data directly comparing RAR with other indicators in terms of cost, ease of measurement, and other specific advantages. Most studies focus on the clinical utility and value of these indicators in predicting the prognosis of specific diseases. However, RAR is an easily obtainable and inexpensive indicator.

**10.Response to comment:** Ambiguous Statements and inconsistent terminology

**Response:** After revision, the expression in parts of the article has been adjusted, and terms have been modified to maintain consistency.

**11.Response to comment:** Some baseline characteristics (e.g., ALT, PTT) show significant differences across groups in Table 1, but the article does not discuss their potential influence. It is recommended to address the potential biases introduced by these differences.

**Response:** We used the Propensity Score Matching method to reduce the imbalance of baseline characteristics between groups, thereby avoiding the impact of these imbalances on the outcomes. We conducted Kaplan-Meier analysis and constructed COX regression models using data from before and after PSM, respectively, and the results from the two groups were consistent.

**12.Response to comment:** Reliance on a single database (MIMIC-III) limits the generalizability of the results. Mentioning the need for future validation with independent datasets in the discussion would strengthen the study.

**Response:** The data in this study is solely derived from the MIMIC III database, which limits the generalizability of the results. As Reviewer suggested, in the limitations section of the discussion, we mentioned that the necessity of validating the findings using other datasets in the future is required to strengthen the results of this study.

**13.Response to comment:** The reported AUC of 0.669, though statistically significant, indicates limited predictive ability. This should be acknowledged in the discussion, and the potential for combining RAR with other indicators to enhance predictive power should be explored.

**Response:** In this study, the AUC of RAR is 0.669, indicating limited predictive power. Considering the Reviewer' s suggestion, in the discussion, we have stated that the potential for combining RAR with other indicators to enhance predictive power should be explored.

**14.Response to comment:** The article mentions adjusting for all variables with p < 0.05, but the comprehensiveness and methodology of the adjustments are unclear. For example, was the role of gender and other potential confounders thoroughly addressed?

**Response:** Regarding the selection of adjustment variables, in Model 2, the adjustment variables consist of demographic characteristics, including gender, age, race, and comorbidities. Model 3 builds upon Model 2 by adding variables with a p-value less than 0.05 in the multivariate analysis, which includes potassium, total bilirubin, blood urea nitrogen, lactate, partial thromboplastin time, and neutrophil count. Model 4 further includes the use of invasive mechanical ventilation on basis of the variables included in Model 3, in an effort to control for as many potential confounding factors as possible.

**15.Response to comment:** Ensure that the reference style fully complies with journal requirements.

**Response:**The reference style has been adjusted according to the requirements of the journal.

**16.Response to comment:** The number of references appears to be relatively low. Consider adding citations to fully support the content of the article.

**Response:** The number of references has been appropriately increased to support the content of the article.

Special thanks to you for your good comments.We have tried our best to improve the manuscript and made some changes in the manuscript. The changes have been marked in red in revised paper.We appreciate for your warm work earnestly, and hope that the correction will meet with approval.
