# Supplementary material for: Association between red blood cell distribution width-to-albumin ratio at admission and all-cause mortality in patients with acute pancreatitis based on the MIMIC-III database
Source: PLoS One. 2025 Feb 7;20(2):e0318873. doi: 10.1371/journal.pone.0318873 (PMC11805432; doi:10.1371/journal.pone.0318873)
Supplement: Attachment — Submitted filename: Response to Reviewers.docx [file pone.0318873.s006.docx]

Dear Editors and Reviewers:

Thanks for your continued attention to the manuscript entitled "Association between red blood cell distribution width-to-albumin ratio at admission and all-cause mortality in patients with acute pancreatitis based on the MIMIC-III database"(ID:PONE-D-24-38811). First and foremost, I must extend my sincerest apologies for not adequately addressing the feedback provided by the reviewers. I had incorrectly assumed that detailing responses to each point within the reviewer letter would be adequate. The comments provided are immensely valuable and constructive, offering significant insights for revising and enhancing our manuscript. We have meticulously addressed each comment and have made the necessary corrections accordingly. The revised sections are highlighted in red throughout the paper for ease of identification. Below, we outline the primary corrections made to the manuscript and our detailed responses to the reviewers’ comments:

**1.Response to comment:** The quartile divisions for RAR seem arbitrary. Were alternative methods of stratification (e.g., tertiles, clinical thresholds) considered? If not, justify why quartiles were chosen.

**Response:** I acknowledge that the issue of arbitrary grouping based on quartiles was identified during the initial revision of the article. Initially, the choice to group the data by quartiles was made, which indeed may have seemed somewhat arbitrary. Reflecting on this, I have made a crucial adjustment to the grouping criteria. For this study, I have now adopted the cutoff value of RAR as the grouping criterion, which aligns more closely with the clinical thresholds established in previous research. This change aims to provide a more clinically relevant and justified basis for the groupings in our analysis.

**2.Response to comment:** Subgroup analysis results need further interpretation. The lack of significance in diabetic patients and those with high SAPSII scores should be discussed.

**Response:** Following the regrouping based on the cutoff value of RAR, a subgroup analysis was conducted to explore whether the association between RAR and 28-day mortality varied across different subgroups of patients. The results of this analysis indicated that the RAR did not demonstrate significant interaction effects across all the subgroups examined. This suggests that the relationship between RAR and 28-day mortality is consistent across various patient characteristics.

**3.Response to comment:** I recommend sensitivity analyses should also be performed to test the robustness of findings.

**Response:** To establish the robustness of the association between RAR and 28-day mortality in AP, in addition to adjusting the covariates in the COX model, we separately conducted COX regression analyses to calculate hazard ratios after: (1) excluding extreme values, and (2) stratifying patients into two groups based on the median RAR value. The consistent findings from both the outlier-removed and median RAR-based patient grouping COX regression analyses underscored the robust association between the RAR and the 28-day mortality risk in AP.

**4.Response to comment:** Expand the background on the clinical utility of RAR.

**Response:** In the discussion part, we expanded the background of the clinical application of RAR. Studies have shown that higher baseline RAR is related to the increased risk of all-cause and cause specific mortality in the general population. In addition, RAR may become a valuable prognostic biomarker for patients with coronary heart disease and diabetes in the ICU.

**5.Response to comment:** It will be better to highlight variables with significant differences in table.1.

**Response:** The variables with significant differences have been highlighted in Table 1.

6. **Response to comment:** I recommend that the author considers language polishing to improve clarity and readability. For example, replace "thoroughgoing estimation" with "detailed evaluation".

**Response:** We agree that clear and precise language is crucial for effective communication of our research findings.In response to your suggestion, we have undertaken a comprehensive language polish throughout the manuscript to ensure that the text is both clear and readable.

We appreciate your attention to detail and your contribution to improving the quality of our work.We have tried our best to improve the manuscript and made some changes in the manuscript. The changes have been marked in red in revised paper.We appreciate for your warm work earnestly, and hope that the correction will meet with approval.
